# Cheminformatics Research at the Unilever Centre for Molecular Science Informatics Cambridge

**DOI:** 10.1002/minf.201400166

**Published:** 2015-03-10

**Authors:** Julian E Fuchs, Andreas Bender, Robert C Glen

**Affiliations:** [a] Centre for Molecular Informatics, Department of Chemistry, University of Cambridge Lensfield Road, Cambridge CB2 1EW, UK phone/fax: +44 (0)1223 336472/+44 (0)1223 763076

**Keywords:** Centre for Molecular Science Informatics, Retrospective analysis, History, Trends, Directions

## Abstract

The Centre for Molecular Informatics, formerly Unilever Centre for Molecular Science Informatics (UCMSI), at the University of Cambridge is a world-leading driving force in the field of cheminformatics. Since its opening in 2000 more than 300 scientific articles have fundamentally changed the field of molecular informatics. The Centre has been a key player in promoting open chemical data and semantic access. Though mainly focussing on basic research, close collaborations with industrial partners ensured real world feedback and access to high quality molecular data. A variety of tools and standard protocols have been developed and are ubiquitous in the daily practice of cheminformatics. Here, we present a retrospective of cheminformatics research performed at the UCMSI, thereby highlighting historical and recent trends in the field as well as indicating future directions.

## 1 Introduction

In December 2000 the Unilever Centre for Molecular Science Informatics (UCMSI) was opened at the Department of Chemistry of University of Cambridge. Based on an investment by the industrial partner Unilever, a new world-leading research group in the emerging field of molecular informatics was established. The investment included a new building, an established chair in Molecular Science Informatics and three lectureships as well as set up costs (equipment, networking, and software). The Unilever research grants were renewed in year five and year ten. In addition, over the period of the UCMSI’s existence, significant additional grants from a variety of industrial, charitable and research council sources were obtained to support the objectives of the UCMSI.

The research centre is located in central Cambridge, thus profiting from a stimulating and exciting research environment. Daily interactions with several other local institutes at the University of Cambridge, the EMBL European Bioinformatics Institute (EBI), and the Cambridge Crystallographic Data Centre (CCDC) create a world class research cluster. Furthermore, several major industrial partners are located in close proximity in Cambridge’s science parks and on corporate research sites.

Collaborations with more than twenty industrial partners and especially Unilever allowed access to high quality data sets and formed the basis for state-of-the-art computational modelling.[1] Further industrial partners have included Boehringer Ingelheim, AstraZeneca, BASF, Pfizer, Johnson&Johnson, GSK, Aboca and Eli Lilly, to name but a few. Additional third party funding was attracted at the national level from the UK Engineering and Physical Sciences Research Council, The Medical Research Council, The Wellcome Trust, and the Biotechnology and Biological Sciences Research Council, as well as The National Institutes of Health in the USA. On the European level, major grants from the European Chemical Industry Council (CEFIC) and the European Research Council (ERC), and the Framework-7 program were successfully obtained, funding a variety of international informatics research projects.

The research at the UCMSI covers broad areas of cheminformatics, which coupled with experiments and collaborations with industrial partners in bringing products to the market, ensures real life feedback in several inter-disciplinary research efforts. A main goal of the currently 40 scientists in the UCMSI is the integration of chemistry, biology and materials science through the development and application of molecular informatics. Robert Glen has directed the UCMSI since its opening. He heads an interdisciplinary research group using a broad set of computational methodologies to tackle basic scientific questions in the general area of molecular biosystems. Four additional research groups are focusing on relevant areas on cheminformatics research at the UCMSI. The group of Jonathan Goodman focuses on synthesis, computation and informatics and applies cheminformatics to tackle questions in chemical reactivity and catalysis. Peter Murray-Rust’s group focuses on semantic web technologies and develops methodologies and software for intelligent storage and retrieval of chemical data. Andreas Bender’s group drives the integration of new large scale data sources (gene expression, biological networks, phylogenetics) in the field of cheminformatics. Recently, Lucy Colwell has established a new group at the Centre, her research aims to identify structural features within large datasets using advanced statistical and data analytics methodologies. Two former group leaders have recently departed from the UCMSI to set up significant research groups. Peter J. Bond recently moved on to a principal investigator position at the Bioinformatics (BII) Institute A*STAR in Singapore and continues his research efforts on multiscale modelling and large scale simulations. Former group leader John Mitchell has moved to the University of St. Andrews as a Reader and continues broad research in cheminformatics from quantum chemistry to molecular simulation technologies. Former group leaders include David Lary, Guy Grant, Dmitry Nerukh, Maxim Federov and Hamse Mussa.

Over the years of the Centre’s existence 67 PhDs have been trained and 36 postdoctoral research associates performed high quality research. The UCMSI hosted 50 conferences/workshops and organized over 200 seminars. Scientists at the UCMSI have won several awards and prizes including the RSC Bader Award to Jonathan Goodman, the Hansch Award to Andreas Bender, and the Novartis Chemistry Lectureship to Robert Glen. In this article we describe the developments in cheminformatics research performed at the UCMSI aiming to identify challenges and opportunities for the field in the future.

## 2 Methods

### 2.1 Data Mining in Web of Science

We retrieved all entries related to the UCMSI in all Web of Science databases.[2] Therefore, we used the Web of Science web interface and extracted entries with at least one authors address containing all words “Cambridge”, “University”, “Molecular”, and “Unilever”. Furthermore, we discarded entries in the database earlier than the year 2001, to remove three false positive hits of earlier years. Unfortunately, some entries (e.g.[3,4]) are discarded as false negatives at this stage, as different abbreviations in author affiliations have been used. Citing articles and total citations were extracted for all articles and analyzed in terms of self- and non-self citations according to the tools provided in the Web of Science online mask. The Hirsch index (h-index)[5] was calculated to estimate a total impact of the science performed at the UCMSI.

### 2.2 Analysis of Publications Using Word Clouds

Furthermore, we extracted information on author names and abstracts (where available) from all publications. We identified the leading scientists and their central research topics using word cloud representations (“wordles”) generated using WordItOut.[6] We used author surnames only to ensure consistency between different data sources. Full abstracts were used for the generation of topic-related word clouds. To allow for identification of trends over time we split the complete data sets for authors and abstracts into sections of years. Thereby, we analyzed author and thematic contributions for the years 2001–2004, 2005–2007, 2008–2010, and 2011–2014 respectively.

## 3 Results

**Figure 1:**
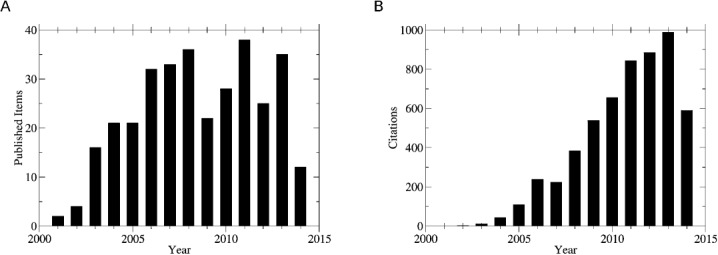
Scientific publications at the UCMSI: A) Published items at the UCMSI are shown over the years of its existence since 2000. An increase to a stable plateau of around two publications per month is visible. B) Citations related to items published at the UCMSI. The increasing importance of research performed in the field of cheminformatics is reflected by steadily growing citations per year.

We identified in total 325 published items of the UCMSI in the Web of Science database. After an initial growth phase, the article output has reached a stable plateau of between twenty and 40 published items per year (see Figure 1A). The observed decrease for the year 2014 arises from incomplete data for the current year. Linear extrapolation to the complete year increases the number of published articles listed in Web of Science to the average level of approximately 20. Due to lagging indexing in Web of Science linear extrapolation is expected to underestimate values for year 2014. When setting aside the incomplete year 2014, Pearson correlation between years and published items is 0.78, indicating a steadily growing output of research performed at the Centre.

In addition to the number of published items, citations were counted on articles published by the Centre. Soon after publication of the first two articles in 2001, first citations are recorded (see Figure 1B). The amount of citations continues to grow steadily over the years and breaks the barrier of 100 citations in the year 2005. Results are shown on a per-year basis, not as a cumulative count. Nevertheless, an almost linear increase of citations with Pearson correlation coefficient of 0.97 between year and citations is observed when excluding the incomplete year 2014. With 988 citations in the year 2013 only, research from the UCMSI is referenced almost three times per day. Linear extrapolation for year 2014 indicates that the barrier of 1000 citations is likely to be broken for the first time.

In total 5508 citations on the 325 articles were recorded via Web of Science. Only nine percent of these represent self-citations (506 citations), thus reflecting a broad audience and considerable impact within the cheminformatics community. 4109 unique indexed articles reference scientific reports from the UCMSI with less than five percent of self-citations (177 articles). On average, articles from the Centre are cited 16.95 times.

No single article accounts for the large average citation count. By contrast, eight articles are individually cited over 100 times, all of them have been published in 2004 or later.[7–14] This broad impact is reflected by an h-index of 39 after 14 years of existence of the Centre. Eight articles are indicated as “highly cited articles” within Web of Science,[9,11–13,15–18] thus representing the top one percent publications in the respective field. These articles only partially overlap with articles already cited more than 100 times. This indicates that they will most likely be the next ones crossing the barrier of 100 citations.

**Figure 2:**
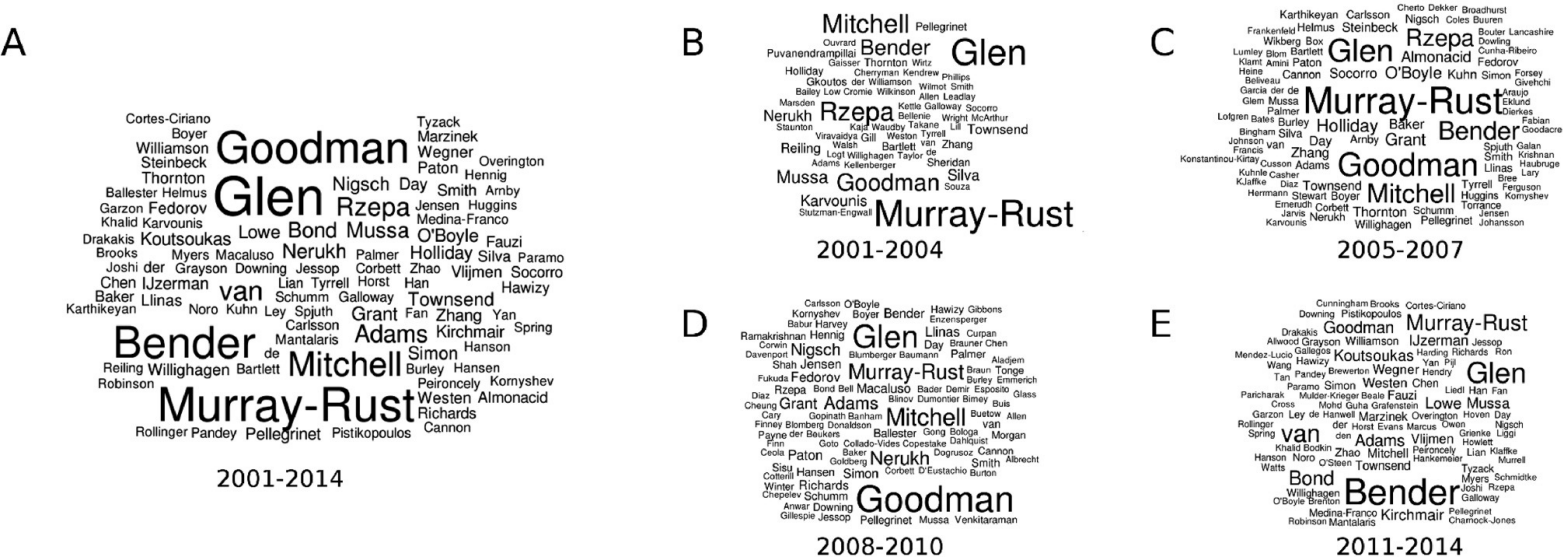
Word clouds of co-authors listed on publications of the UCMSI: A) Word cloud over all years of existence. B–E) Author occurrences are split according to years 2004–2004, 2005–2007, 2008–2010, and 2011–2014 respectively.

By creating word clouds from author lists of all published items we identified key scientists at the UCMSI in Cambridge (see Figure 2A). Robert C. Glen has published 83 items over the years, closely followed by the group leaders Peter Murray-Rust, Andreas Bender, and Jonathan Goodman, each contributing between 60 and 70 articles. Splitting author contributions according to intervals of years allows the development of the UCMSI to be followed. In earlier years, lecturer John Mitchell was a highly active researcher at the UCMSI, as was Henry Rzepa in collaboration with Peter Murray-Rust (see Figure 2B). In later years the name “Andreas Bender” emerges more and more with a short break during his time at Novartis and the University of Leiden (see Figure 2C and Figure 2D). In recent years, the group of Peter Bond focusing on simulations added additional output to the UCMSI (see Figure 2E).

**Figure 3:**
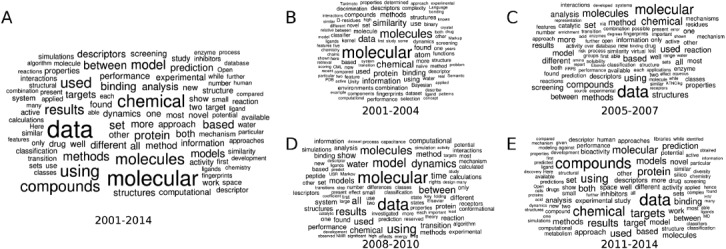
Wordles generated from indexed abstracts of publications from the UCMSI: A) Word cloud over all years of existence of the Centre. B-E) Word occurrences are analysed in terms of chronological development over the years 2001–2004, 2005–2007, 2008–2010, and 20011–2014.

Wordles created on the basis of published abstracts identify key topics and methods applied at the UCMSI (see Figure 3A). “Data” is the most abundant single phrase and appears mostly in connection with the second most occurring words “molecular” and “chemical”. Therefore, chemical data form the foundation of all research performed at the Centre. “Model” and “models” appear prominent in the list, giving hints how chemical data is utilized in the generation of computational models for a variety of mostly chemical and biological properties.

Splitting abstracts according to publication years does not reveal any consistency in the research efforts on-going at the UCMSI (see Figures 3B–3E). Contributions of “molecular” and “data” are constantly high. Some words linked to the observables predicted in established models appear transiently, e.g. “target” for recently established target prediction tools. Nevertheless, the low frequency of individual modelled molecular properties reflects the broadness of topics covered. Furthermore, structure-based modelling approaches[19] appear more prominent in recent years as indicated by the increasing occurrence of the keywords “protein”, “water”, and “dynamics”.

## 4 Discussion

Analysis of citations has allowed the identification of key topics and methods applied at the UCMSI. Science is often centred around method development which is reflected by several publications in leading journals in the field of cheminformatics and modelling. Additionally, application of the newly developed methods ensures real life feedback and enabled publications in fields ranging from drug discovery to synthesis, materials science to electrochemistry. In the following paragraphs we will select particular fields of research where major advances have been made at the Centre.

### 4.1 Linking Cheminformatics and Biology

**Figure 4:**
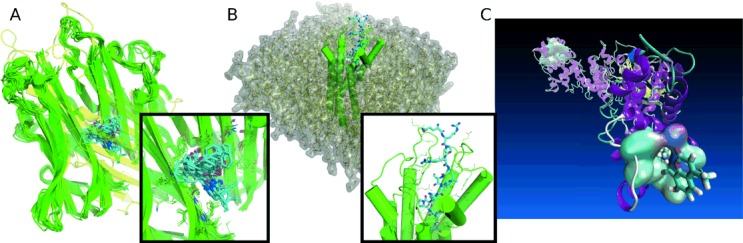
Examples of cheminformatics research currently performed at the UCMSI: A) Ensemble of protein-ligand poses extracted from a molecular dynamics simulation of tumor necrosis factor α (TNFα, green cartoon) in complex with a biscoumarin (cyan sticks). The native trimeric structure of TNFα (semi-transparent yellow cartoon, PDB: 1TNF) is disrupted by ligand binding. A zoom on the binding site highlights the hydrophobic environment of the ligand, allowing for several binding modes. B) Model of the apelin receptor (green cartoon) in complex with the apelin-13 peptide (cyan sticks). Starting coordinates of a molecular dynamics simulation box are depicted including a model lipid bi-layer (yellow lines and semi-transparent surface) along with explicit water molecules and counter ions (both not shown). The predicted binding mode of the apelin-13 peptide and surrounding residues are highlighted in the included zoom on the binding cavity. C) Illustration of the simulation approach followed to investigate selectivity of Schiff base forming covalent IRE-1 inhibitors. IRE-1 is shown as cartoon with the reactants Lys-907 and inhibitor 4µ8C highlighted as sticks.

Starting from innovative ways to encode and compare chemical environments in molecules,[7,8,10] several steps have been taken to link chemical with biological properties of molecules[20] based on statistical modelling techniques.[21,22] Thereby, the fields of cheminformatics and bioinformatics increasingly overlap and even fuse, leading to approaches like proteochemometric modelling (PCM).[23,24] With the establishment of target prediction tools based on statistical modelling,[25,26] novel approaches to cluster molecules using predicted biological effect can be implemented.[27] Furthermore, these novel methodologies proved to be helpful in multi target drug design.[28] Recent directions in the area comprise the inclusion of further biological data sources, e.g. from biological networks and phylogenetics[29,30] as well as gene expression data.[31] Several successful applications of target prediction algorithms[32–34] underline the increasing accuracy of such data-driven approaches and point towards a bright future given the increase in available data sources.[35,36] In a recent success story cheminformatic tools were applied to identify potential targets of a series of synthetic biscoumarins showing anti-cancer activity in vitro and in vivo.[37] After in depth computational characterization of predicted protein-ligand interactions (see Figure 4A), the in silico predicted protein target tumour necrosis factor α was verified by experimental techniques. An emerging future direction in this field is the prediction of biological effects of compound combinations that is financed via an ERC Starting Grant to Andreas Bender. Further stimuli in this area expected from statistical analyses of genomic sequence data.[38,39]

### 4.2 Data Semantics and Accessibility

Growth of the available (“Big”) data brings new challenges to the field of cheminformatics. Millions of chemical substances have been characterized, many hundreds of thousands of three-dimensional structures have been deposited in databases, and associated biological data is spread over millions of publications and patents.[40] Therefore, the development of standards for chemical identification, indexing and storage is crucial for future successful data retrieval. Usage of the IUPAC standard International Chemical Identifiers (InChIs) allows the encoding of chemical information using a unique text descriptor, e.g. for database searches.[41] Based on InChIs several tools have been developed, e.g. to convert structures to chemical names in a fully automated way[42] or chemistry-aware text mining.[43] Recently, InChIs have been extended to RinChIs to depict chemical reactions unambiguously.[44] Chemical Markup Language (CML) introduces and specifies particular data fields to efficiently store and retrieve chemical data.[45] Based on CML the World-Wide Molecular Matrix (WWMM) has been introduced to collect and connect chemical information of various sources.[46] Chem4Word has been created in a collaboration between Microsoft Research and the UCMSI to facilitate the handling of chemical information within text processing software. To date, the plug-in has been downloaded more than 400 000 times. Over the last decade, the UCMSI has been a key player in setting standards for open data and data quality standards in chemistry.[47–49] In recent years, technologies for data semantics and natural language processing have been employed attempting to directly extract the scientific context of published chemical data.[50]

### 4.3 Modelling of Physicochemical Properties and ADMET Parameters

The increase in data sources over recent years allowed the establishment of more accurate computational models, even for complex biological phenomena, e.g. absorption, distribution, metabolism, excretion and toxicity (ADMET) of xenobiotics.[51] Dozens of computational methods for prediction of metabolic reactions on different levels of complexity have been published in the literature.[16] Thorough classification of annotated biotransformations[52] facilitated the development of novel predictive models for general metabolic reactivity (Metaprint2D),[53,54] P-glycoprotein transport,[55] solubility,[56] and cytochrome P450-catalyzed metabolic reactions.[57–59] Thereby, innovative computational methods making use of recent advances in graphics processing unit (GPU) based computing have been employed. Using these approaches, new levels of throughput and thus modelling accuracy are in range. Furthermore, the UCMSI ran a solubility competition (with over 100 entries) for the cheminformatics community and provided high quality experimental data to facilitate further method developments in the area.[60–62]

### 4.4 Bioactive Compound Discovery

The UCMSI has been a driving force of computer-aided drug design over the past decades. In addition to support of external drug design efforts, local lead discovery projects have been guided by computational technologies. Novel ligands for the G-protein coupled receptor (GPCR) apelin have been successfully identified and biologically characterized.[63] Based on modelled structures of the apelin receptor (see Figure 4B), optimization of the peptide-derived compounds is on-going and shows enormous potential for further development and recently the first human study of apelin biased agonists has been completed in Addenbrooke's hospital in Cambridge. (Design, characterization and first-in-human study of the vascular actions of a novel ‘biased’ apelin receptor agonist. Anthony Davenport, Aimee Brame, Janet Maguire, Peiran Yang, Alex Dyson, Rubben Torella, Joseph Cheriyan, Mervyn Singer, Robert Glen, Ian Wilkinson. Hypertension, in press, 2015). Furthermore, small molecule antagonists of the 5-HT1B GPCR have been identified and optimized for development as a potential treatment for Pulmonary Hypertension.[64] Several newly designed and synthesized compounds are currently in clinical studies in Addenbrooke’s Hosptial, Cambridge. In another compound discovery effort large scale simulation approaches (see Figure 4C) have been used to identify covalent binders of the endonuclease domain of IRE-1.[65,66] In a follow-up study selectivity of the Schiff base forming compounds has been investigated by advanced computational techniques.[67] Research at the UCMSI also lead to joint patents with Unilever on the CB1/2 receptors and NCKX ion channels showing benefits in skin for use in home and personal care products. To assist drug discovery several analyses of molecular diversity in the context of diversity-oriented chemical synthesis have been performed in collaboration with experimental groups.[68–70]

In addition to these four areas the UCMSI has been particularly active in development of innovative simulation and analysis methodologies[71,72] and cheminformatic support for organic synthesis.[73,74] Industrial partners emphasize the productivity of collaborative research efforts with the Centre for Molecular Informatics. Ola Engkvist, team leader of Computational Chemistry at AstraZeneca and involved in several joint research projects, highlights: “The collaboration with the UCMSI provides AstraZeneca with the opportunity to work closely with one of the world leading groups in cheminformatics. The combination of the UCMSI’s outstanding scientific knowledge with AstraZeneca’s industrial experience provides a platform for state-of-the-art research in cheminformatics. The proximity to one of the AstraZeneca science hubs is an additional plus that facilitates smooth collaborations.” Jim Crilly, senior vice-president of the Strategic Science Group at Unilever, adds: “Unilever is very proud to have instigated in partnership with the University of Cambridge the Centre for Molecular Informatics under the Leadership of Professor Robert Glen and pleased that it has developed into a global centre of excellence in a hugely important field of research. Collaboration with the centre has brought a new way of working into our own research endeavours and accelerated our discovery process.”

Following a clear mission statement published in 2002[75] the UCMSI is developing tools and standards in molecular informatics. With the increase in computer power and data accessibility, molecular modelling allows chemical and biological effects of increasing complexity and size to be captured. Available data sources range from chemistry and related bioactivity (PubChem,[76] ChEMBL,[35] DrugBank[77]) via protein sequence (UniProt[78]) and structure (Protein Data Bank,[79] Pfam[80]), to cellular pathways (KEGG[81]) and responses (LINCS[82]). A collection of online molecular biology databases has recently been published with the database issue of Nucleic Acids Research.[83]

With the advent of microsecond simulations of biological macromolecules[84] and sophisticated enhanced sampling methods,[85] dynamic processes underlying macromolecular recognition processes can be modelled with a new level of accuracy.[86] Protein-ligand binding processes may be studied at atomistic resolution, reports include full sampling of binding and unbinding of both fragments[87] and small molecules.[88] With an increasingly accurate description of protein dynamics, its role in biomolecular recognition processes can be studied, thereby allowing pharmaceutically relevant properties like binding specificity[89] or binding kinetics[90] including allosteric mechanisms[91] to be probed. On the other hand, integration of innovative data sources from emerging “omics” fields such as proteomics,[92] metabonomics/metabolomics,[93] or lipidomics[94] will allow the capture of novel biological properties which have limited or no direct chemical leads to their mechanism and function.

## 5 Conclusions

The data presented underline the central role in the cheminformatics world that is occupied by the UCMSI. The research institute sets world-wide standards and publishes technologies and software that is widely used and cited. The broad range of topics covered at the UCMSI ensures its broad impact ranging from basic cheminformatics, data mining and machine learning techniques to complex simulations, to chemical reactivity and synthesis. The connection of all these areas in a single research centre offers unique opportunities for scientists involved, industrial partners, as well as the whole cheminformatics community.
